# Fabrication of an Organofunctionalized Talc-like Magnesium Phyllosilicate for the Electrochemical Sensing of Lead Ions in Water Samples

**DOI:** 10.3390/nano12172928

**Published:** 2022-08-25

**Authors:** Chancellin Nkepdep Pecheu, Sherman Lesly Zambou Jiokeng, Arnaud Kamdem Tamo, Giscard Doungmo, Ingo Doench, Anayancy Osorio-Madrazo, Ignas Kenfack Tonle, Emmanuel Ngameni

**Affiliations:** 1 Electrochemistry and Chemistry of Materials, Department of Chemistry, University of Dschang, Dschang P.O. Box 67, Cameroon; 2 Institut für Anorganische Chemie und Strukturchemie, Heinrich-Heine-Universität Düsseldorf, 40204 Düsseldorf, Germany; 3 Laboratoire de Chimie Physique et Microbiologie pour les Matériaux et l’Environnement (LCPME), UMR 7564 CNRS—Université de Lorraine, 405, rue de Vandœuvre, 54600 Villers-lès-Nancy, France; 4 Laboratory for Bioinspired Materials BMBT, Institute of Microsystems Engineering IMTEK-Sensors, University of Freiburg, 79110 Freiburg, Germany; 5 Freiburg Center for Interactive Materials and Bioinspired Technologies FIT, University of Freiburg, 79110 Freiburg, Germany; 6 Freiburg Materials Research Center FMF, University of Freiburg, 79104 Freiburg, Germany; 7 Institut für Anorganische Chemie, Christian-Albrechts-Universität zu Kiel, Max-Eyth-Str. 2, 24118 Kiel, Germany; 8 Laboratory of Analytical Chemistry, Department of Chemistry, University of Yaounde 1, Yaoundé P.O. Box 812, Cameroon

**Keywords:** sol–gel processing, talc-like clay, amino-functionalized clay, glassy carbon electrode, Pb(II) detection, water bioremediation

## Abstract

A talc-like magnesium phyllosilicate functionalized with amine groups (TalcNH_2_), useful as sensor material in voltammetry stripping analysis, was synthesized by a sol–gel-based processing method. The characterizations of the resulting synthetic organoclay by scanning electron microscopy (SEM), X-ray diffraction, N_2_ sorption isotherms (BET method), Fourier transform infrared spectroscopy (FTIR), CHN elemental analysis and UV–Vis diffuse reflectance spectroscopy (UV–Vis-DRS) demonstrated the effectiveness of the process used for grafting of amine functionality in the interlamellar clay. The results indicate the presence of organic moieties covalently bonded to the inorganic lattice of talc-like magnesium phyllosilicate silicon sheet, with interlayer distances of 1568.4 pm. In an effort to use a talc-like material as an electrode material without the addition of a dispersing agent and/or molecular glue, the TalcNH_2_ material was successfully dispersed in distilled water in contrast to natural talc. Then, it was used to modify a glassy carbon electrode (GCE) by drop coating. The characterization of the resulting modified electrode by cyclic voltammetry (CV) and electrochemical impedance spectroscopy (EIS) revealed its charge selectivity ability. In addition, EIS results showed low charge transfer resistance (0.32 Ω) during the electro-oxidation of [Fe(CN)_6_]^3−^. Kinetics studies were also performed by EIS, which revealed that the standard heterogeneous electron transfer rate constant was (0.019 ± 0.001) cm.s^−1^, indicating a fast direct electron transfer rate of [Fe(CN)_6_]^3−^ to the electrode. Using anodic adsorptive stripping differential pulse voltammetry (DPV), fast and highly sensitive determination of Pb(II) ions was achieved. The peak current of Pb^2+^ ions on TalcNH_2_/GCE was about three-fold more important than that obtained on bare GCE. The calculated detection and quantification limits were respectively 7.45 × 10^−8^ M (S/N = 3) and 24.84 × 10^−8^ M (S/N 10), for the determination of Pb^2+^ under optimized conditions. The method was successfully used to tap water with satisfactory results. The results highlight the efficient chelation of Pb^2+^ ions by the grafted NH_2_ groups and the potential of talc-like amino-functionalized magnesium phyllosilicate for application in electrochemical sensors.

## 1. Introduction

Talc or steatite is a clay mineral with the chemical formula Mg_3_Si_4_O_10_(OH)_2_. It consists of tetrahedral Si and octahedral Mg sheets forming the layered structure 2:1 [1,2]. Silicon dioxide (SiO_2_), magnesium oxide (MgO) and H_2_O are the main constituents of talc [3]. The talc surface consists of basal cleavage faces and edges. The neutral surface faces consist of tetrahedral siloxane with -Si-O-Si- bonds, giving them a non-polar and hydrophobic character, while the edges are hydrophilic due to the presence of charged ions (Mg^2+^ and OH^−^) [4]. The hydrophobic behaviour of the surface layers of talc is due to oxygen atoms [5]. Talc has very interesting properties, it is organophilic, has a lamellar structure, chemical inertia, high thermal stability, low electrical conductivity, heat resistance, wide particle size distribution and high specific surface area [6,7]. Due to its hydrophobic nature, talc is highly valued for various applications such as paints, adhesives and sealants [8]. Despite its good properties, its chemical reactivity is limited when it has to undergo chemical surface modification with organic molecules, due to the rather strong attractive forces between the sheets [9]. Previous studies have shown that talc can be easily dispersed in a surfactant/polymer by absorption on its surface [10]. However, its difficult dispersion in aqueous solution limits its applications, for example, in the development of electrochemical sensors [11]. 

Synthetic clay materials are very popular compared to their natural counterparts due to their high purity [12,13]. Inorganic–organic materials based on magnesium silicates, similar to natural talc, have been synthesised by a sol–gel process. Under normal conditions of temperature and pressure, covalent attachment of organic functionalities occurs, thus creating homogeneous pure inorganic-organic hybrid materials with controlled porosity [14]. Ca^2+^ and Ni^2+^ ions have been extensively studied to obtain these organosilicate materials [15,16,17,18]. However, other organomodified phyllosilicates have also been synthesised, including aluminium [15], copper [19], zinc [20], and calcium [21] inside the inorganic layer. It should be noted that the magnesium and nickel-based organosilicates have a lamellar structure similar to the 2:1 trioctahedral phyllosilicates. The main objective of these synthetic materials remains their applicability, which would be multiple in case of organo-functionalisation [22]. Organoclays can be used as adsorbents, environmental barriers, polymer fillers, catalytic supports, electrochemical sensors or chemical sensors [23]. Previous work has shown their use for binding cations in aqueous solutions at the solid/liquid interface [16,19,20,24]. Their ability to extract heavy metals is a promising property to be explored in many applications [25].

Lead is a heavy metal that can have negative effects on human health, including kidney disease, cardiovascular effects, reproductive toxicity and irreversible nerve damage [26,27]. The Quantification of lead in real samples is usually performed by electrothermal atomic absorption spectrometry [28], atomic fluorescence spectrometry [29], inductively coupled plasma spectrometry [30]. As these methods are somewhat limited in selectivity and sensitivity, electrochemical methods have been widely studied as electrochemical sensors [31,32]. In this regard, several works based on chemically modified electrode materials have been proposed [33,34,35,36,37,38]. Most of these studies demonstrate organoclays for heavy metal electroanalysis due to the affinity between the organic modifier and the target species. To our knowledge, no work in the literature mentions the use of amino-functionalized magnesium phyllosilicates of the synthetic talc type as a glassy carbon electrode (GCE) modifier.

This work describes the preparation of a new amino-functionalised magnesium phyllosilicate obtained by a sol–gel process. The resulting synthesised material and natural talc were characterised by physicochemical methods. The characterised material was tested as electrochemical sensor with electrochemical characterizations of the modified electrode, which was tested for the detection of Pb^2+^ ions in aqueous solution, by means of anodic stripping differential pulse voltammetry (DPV).

## 2. Materials and Methods

### 2.1. Chemicals

The natural talc clay mineral (Nat-Talc) used in this work is a commercial sample (NICRON^®^ 674, Luzenac America, Inc., 767 Old Yellowstone TrI, Three Forks, MT, USA). All chemicals were obtained commercially and used without further purifications. These included (3-aminopropyl)triethoxy-silane (APTES, 99%, Sigma-Aldrich Taufkirchen, Bavaria, Germany), MgCl_2_·6H_2_O (99%, Fluka, Buchs, Switzerland), Pb(NO_3_)_2_ (99%, Analar, Princeton, NJ, USA), K_3_[Fe(CN)_6_] (Prolabo, Bern, Switzerland) and Ru(NH_3_)_6_Cl_3_ (Alfa, Binfield, UK). NaNO_3_ (99.99%, Prolabo, Bern, Switzerland), KCl (99.5%, Fisher Scientific International Inc., Pittsburgh, PA, USA), HCl (36%, Phillip Harris, Birmingham, England), NaCl (99.5%, Fisher Scientific International Inc., Pittsburgh, PA, USA). Zn(NO_3_)_2_·6H_2_O (98%), Cd(NO_3_)_2_·4H_2_O (98%), Cu(NO_3_)_2_·xH_2_O (99.99%) and Hg(NO_3_)_2_·H_2_O (99.99%) were from Sigma-Aldrich (Taufkirchen, Bavaria, Germany).

### 2.2. Material Characterization 

In order to evaluate the morphology of our materials, an Amray 1610 Turbo scanning electron microscope (SEMTech Solutions, Inc., North Billerica, MA, USA) was used. For the measurements, the samples were deposited on a conductive strip placed on a specimen holder and coated with gold by vacuum sputtering. 

Characterisation by Fourier Transform Infrared Spectroscopy (FTIR) was carried out using a Nicolet 8700 instrument equipped with a specular reflectance accessory (Smart Collector).

The crystallinity of the materials was assessed by X-ray diffraction analysis, using an STOE Stadi-p X-ray powder diffractometer (STOE & Cie GmbH, Darmstadt, Germany) operated at 40 kV and 30 mA with Cu Kα1 radiation (λ = 1.54056 Å), in transmission geometry with an IP-PSD (STOE & Cie GmbH, Darmstadt, Germany) and/or a DECTRIS^®^ MYTHEN 1K detector (DECTRIS, Baden-Daettwil, Switzerland). 

The Brunauer-Emmett-Teller (BET) specific surface areas of the samples were determined by means of N_2_ adsorption-desorption at 77.13 K using a micrometrics model sorptometer (Thermo Electron Corporation, Sorptomatic Advanced Data Processing, Waltham, MA, USA). Before N_2_ adsorption, the samples were degassed at 307.13 K under vacuum. The linear part of the BET equation was used to evaluate the surface area.

A CHNS Euro EA 3000 analyser (HEKAtech GmbH, Wegberg, Germany) was used to determine the chemical composition of our materials.

The optical properties of our materials were determined using a Shimadzu UV-Vis 3101PC Diffuse Reflectance Absorption Spectrophotometer (DRS) in the wavelength range 200–800 nm. BaSO_4_ was used as the reflectance standard.

Voltammetry measurements were performed on a µ-Autolab potentiostat running GPES software and using a standard three-electrode cell (bare or modified GCEs used as working electrodes (WE), the saturated silver chloride electrode (Ag/AgCl/KCl) as reference electrode (RE), and a stainless-steel bar as auxiliary electrode). Cyclic voltammograms of [Fe(CN)_6_]^3−^ and [Ru(NH_3_)_6_]^3+^ ions were recorded from −0.15 V to 0.7 V and from −0.6 V to 0.2 V respectively, in 0.1 M KCl at a scan rate of 50 mV/s, unless otherwise stated and without stirring. Using anodic stripping differential pulse voltammetry (ASDPV), the electroanalysis of Pb^2+^ ions involved two successive steps: open circuit preconcentration of the analyte under gentle agitation followed by voltammetric detection in the potential range of −0.7 V to −0.3 V after 30 s electrolysis at −0.8 V. Electrochemical impedance spectroscopy (EIS) measurements were performed on a Palmsens3 potentiostat driven by PS Trace 4.2 software. It was performed over the frequency range of 0.01 Hz to 10 kHz with a potential amplitude of 10 mV in a 0.1 M KCl solution containing 1 mM [Fe(CN)_6_]^3−^/[Fe(CN)_6_]^4−^. 

#### 2.2.1. Preparation of the Organofunctionalized Talc-like Magnesium Phyllosilicate

The synthetic organic/inorganic hybrid was synthesised according to a previously published procedure [13,39]. Firstly, 845.77 mg of magnesium chloride hexahydrate was dissolved at room temperature under stirring in 100 mL of distilled water. To this solution, a 1.0 mL ethanolic solution containing 1197.6 mg of (3-aminopropyl)triethoxysilane was added dropwise at room temperature. The mixture obtained corresponded to a Si/Mg molar ratio of 4/3, as in natural talc. The resulting dense, pale suspension was placed under stirring for 1 h, and 20 mL of 0.1 M NaOH was added dropwise. The suspension was aged for 24 h at room temperature, then filtered and washed with ethanol and distilled water to neutral pH. After centrifugation, the product obtained was dried under vacuum for 48 h at 50 °C and noted TalcNH_2_. 

#### 2.2.2. Preparation of the Working Electrode

For the electrochemical characterisations, a GCE was used. This was first polished with alumina powder of different sizes, then placed in a 1:1 ethanol-water solution and ultrasonicated for 10 min to remove the remaining alumina particles. GCE modified with a TacNH_2_ film was prepared by drop coating: 6 µL of TalcNH_2_ suspension previously prepared by dispersing 5 mg of TalcNH_2_ in 1 mL of water were drop coated on the active surface of the GCE (about 0.071 cm^2^). The modified electrode was placed in an oven set at 110 °C for 4 min to dry. The modified electrode obtained was TalcNH_2_/GCE.

## 3. Results and Discussion

### 3.1. Physicochemical Characterization of Organofunctionalized Clay Material

Elemental analysis carried out with a CHNS Euro EA 3000 analyser on both natural talc (Nat-Talc) and the synthesised material TalcNH_2_ gave the experimental results shown in Table 1. The theoretical results are also presented in Table 1. During the synthesis of TalcNH_2_, the ethoxy groups of the organosilane molecules were transformed into silanol by hydrolysis, being able to lose an H^+^ proton in basic medium. By being negatively charged, these silanols aggregate into ordered molecular networks as do anionic surfactants [40]. The negatively charged micelles can act as a matrix and attract magnesium cations from solution to give a hybrid layered structure, in which the inorganic phase is formed by Si-O-Mg bonds. For TalcNH_2_ (Table 1), the theoretical contents of CHN meet the experimental results, which correspond to the following formula of synthetic amine talc: Mg_1.8_Si_0.9_O_19_C_6_H_24_N_1.8_. From this formula, it was evident that the presence of nitrogen containing fractions and the obtained C/N molar ratio (3.33) were high and close to the calculated value (3.00), evidence that the organic fractions remained intact during the synthesis process. The high calculated value may be due to partial condensation, involving the methoxy groups of the silylating agents, which would have resulted in an increase in carbon content. The Si/Mg ratio of 0.50 for the TalcNH_2_ material differs from the expected value of 1.33 for natural talc. Clearly, there is 2.67-fold more magnesium in the TalcNH_2_ structure than in natural talc, which could be due to the presence of exchangeable cations between the layers or complexed by the pendant groups arranged in the lamellar cavity. The latter is the most plausible reason as the nitrogen atoms in the pendant groups can complex them [13]. 

**Figure 1:**
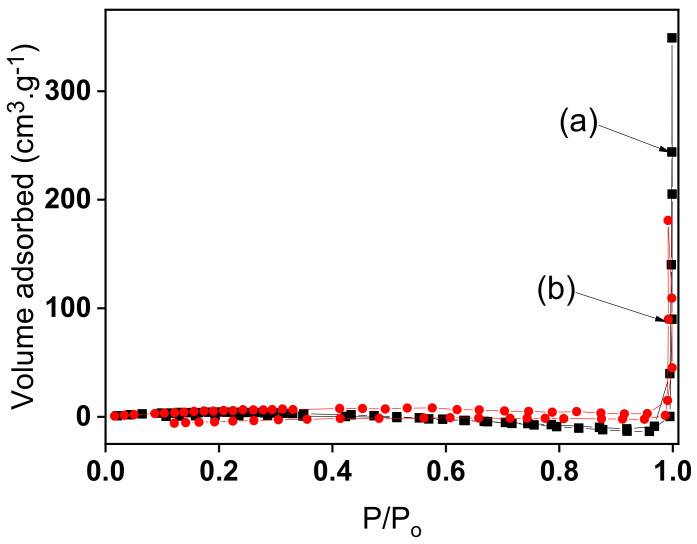
N_2_ sorption isotherms (at 77.13 K) for (**a**) Nat-Talc, and (**b**) TalcNH_2_ materials.

Figure 1 displays nitrogen sorption isotherms for Nat-Talc (curve a) and TalcNH_2_ (curve b). Both clay samples show type III nitrogen isotherms, which is characteristic of non-porous or macroporous solids. A specific surface of 14.39 m^2^/g was obtained for natural talc. This low value compared to those of other families of clay minerals is due to the fact that N_2_ molecules cannot access the interlayer regions of expanding clays or the structural tunnels of natural talc. The cumulative mesopore volume, cumulative micropore volume and surface area, and specific surface area of aminated synthetic talc were 0.013 cm^3^/g, 0.0048 cm^3^/g, 9.04 m^2^/g, and 12.16 m^2^/g, respectively. The low surface area compared to that of natural talc can be attributed to the high level of amino-functionalization of the hybrid material, as the pendant carboxyl groups of the (3-aminopropyl)triethoxy-silane (APTES) molecule block the access of nitrogen gas to the pores of the material [13,41].

**Figure 2:**
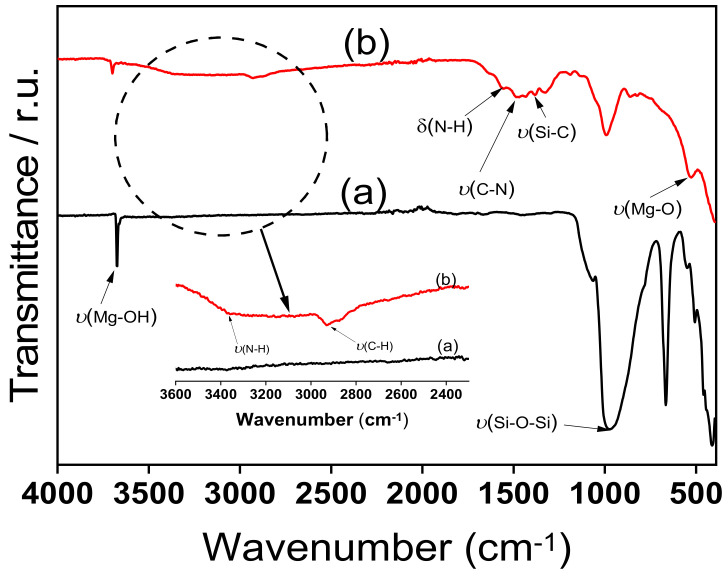
Fourier transform infrared (FTIR) spectra of: (**a**) Nat-Talc, and (**b**) TalcNH_2_.

Figure 2 shows the FT-IR spectra of the raw and synthesised amine clay samples. In the spectrum of raw clay mineral (Figure 2a), a broad band at 3683 cm^−1^, associated with (Mg-OH) vibrations, is observed [42,43]. In addition, some remarkable bands related to the inorganic structure of clay were obtained at 983 cm^−1^ and 535 cm^−1^, due to v (Si-O-Si) and δ(Mg-OH) vibrations, respectively. Some changes were observed in the spectrum of the synthesised clay sample (TalcNH_2_) (Figure 2b). The absorption bands at 3366 cm^−1^ *v*(N-H), 2919 cm^−1^ *v*(C-H), 1613 cm^−1^ δ(N-H), 1481 cm^−1^ *v*(C-N), 1198 cm^−1^ *v*(Si-C), and at 995 and 877 cm^−1^ associated with Si-O-Si and Si-O-Mg bonds [44,45,46,47,48,49]. Around 3600 cm^−1^, we observed a broad band attributed to the vibration of the Mg-OH bond, and the stretching bands of water and the Si-OH bond [50,51,52]. The band at 1628 cm^−1^ corresponds to the bending vibration mode of water. At 535 cm^−1^, another band is observed which is attributed to the overlapping of the Si-O and Mg(OH) vibrational modes as observed in magnesium-based and trioctahedral clays [53,54,55]. However, the presence of new bands observed for the synthetic amino clay corresponds to all the vibrations of the surface organosilane functions. This significant difference shows that the functionalization of the synthetic clay by the APTES molecules was effective.

**Figure 3:**
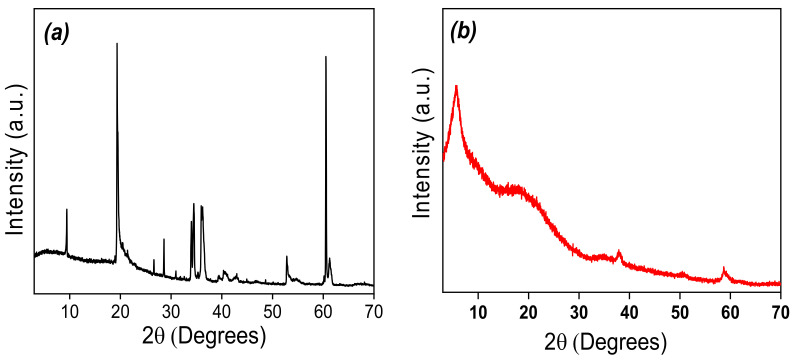
X-ray diffraction patterns of: (**a**) Nat-Talc, and (**b**) TalcNH_2_.

XRD characterizations were performed to compare the crystalline structures of the synthetic and the natural clay materials. XRD patterns of Nat-Talc and synthetic amino-clay (TalcNH_2_) are shown on Figure 3. Figure 3a showed diffraction peaks at 2θ = 9.3° (lamellae width/ spacing d = 950.2 pm), 19.4° and 28.7° that correspond to the primary diffractions of the (001), (020) and (003) planes of natural talc material [56]. The XRD analysis revealed the presence of small amounts of chlorite at 2θ = 26.7° and of dolomite at 2θ = 31°, in addition to talc signals. TalcNH_2_ material showed diffraction peaks at 2θ = 5.63° (lamellae width/spacing d = 1568.4 pm) and at 21.13°, assigned to the primary diffractions of the (001) and (020) planes. The use of (3-aminopropyl)triethoxysilane (APTES) during the synthetic process led to an increase (1.65-fold) in basal spacing when comparing the natural talc value to that of the functionalized talc (950.2 pm to 1568.4 pm, respectively) (Figure 3b). This slight difference arises from the incorporation of organic chains (APTES) in the interlamellar space. This conclusion is derived from the data on the length of the organic chains. By estimating this length using a bond distance model and assuming a zigzag conformation, a value of 543 pm was obtained, for the -(CH_2_)_3_NH_2_ fragments attached to the inorganic backbone. The low intensity peak at 2θ at 59.07° (156.3 pm) corresponds to a reflection in the 060 plane and is in agreement with the formation of trioctahedral layers [15].

**Figure 4:**
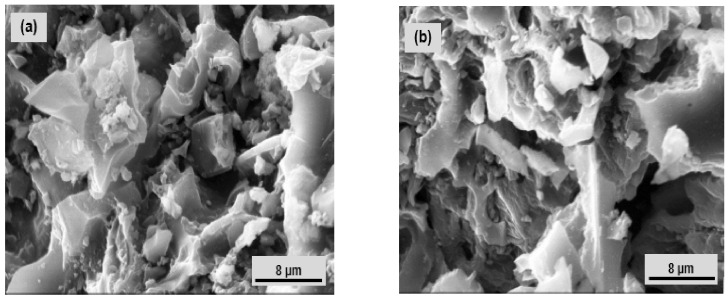
SEM micrographs of: (**a**) Nat-Talc, and (**b**) TalcNH_2_.

Figure 4 shows SEM micrographs of Nat-Talc and TalcNH_2_. The surface morphology of both materials shows a tendency towards aggregation with the microcrystalline character of talc. These observations allowed us to conclude that the structure of Nat-Talc and TalcNH_2_ is practically the same. The raw talc sample showed a solid microporous structure. The surface of natural talc is mainly made up of aggregates or agglomerates of particles with non-uniform pores either in slits, plates or sharpened particles. After functionalization, the synthesised amine talc retained the same surface shape, with a reduction in textural microporosity probably due to the progressive diffusion of APTES molecules between its pores. 

**Figure 5:**
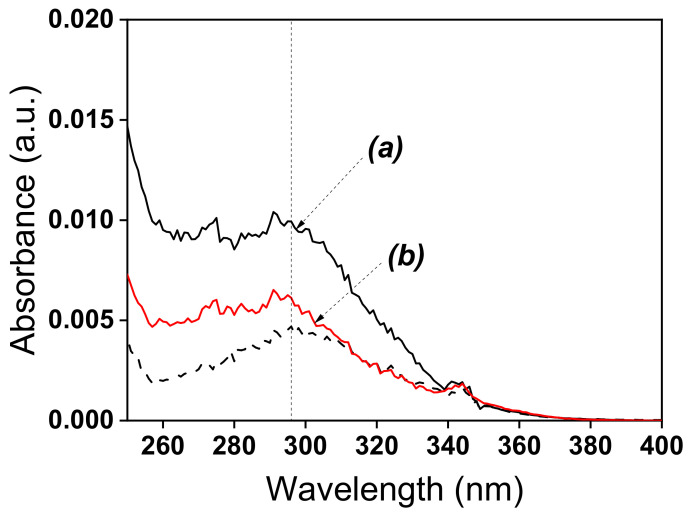
UV-Vis diffuse reflectance spectra of: **(a**) Talc, and (**b**) TalcNH_2_. The dot line corresponds to the reflectance standard BaSO_4_.

The optical properties of raw talc and synthetic amine clay have generally been elucidated by UV-Visible Diffuse Reflectance Spectroscopy (Figure 5). It provides direct evidence of the transmission, absorption or reflection of light by a material. The UV-Vis spectra showed the strongest bands for two materials (Nat-Talc and TalcNH_2_) around 300 nm, indicating that they reflect light in this range. The absorbance values decreased from Nat-Talc (0.01 a.u.) to TalcNH_2_ (0.006 a.u.). The decrease in absorbance is probably related to the incorporation of organosilane into TalcNH_2_ during the synthesis process and to the reduction of the physical surface area of the material. The light thus interacts with the amino parts of TalcNH_2_, highlighting their energy absorption capacity. 

In summary, the presence of C, H, N in the TalcNH_2_ material successfully demonstrated the covalent grafting of organosilane groups onto the chemical functional groups of natural talc, results confirmed by infrared spectroscopy showing the main characteristic bands of APTEAS embedded in the tetrahedral silica layers and by the increase in interlayer distances observed on X-ray diffractograms. Based on the literature on talc and considering the physico-chemical characteristics carried out on the virgin talc used in this work, we noticed that this mineral clay is made up of neutral layers stacked on top of each other and connected by van der Waals interactions. The basal faces of the talc do not carry any -OH functional groups or active ions, while the lateral faces carry very few -SiOH and MgOH functions. The latter two chemical functions mentioned behave as Brönsted acids and are more reactive, whereas the basal surface of talc, consisting of Si-O-Si siloxane bonds, has a low Lewis basicity. Due to the chemical composition as well as the lamellar geometry of talc, which gives talc a hydrophobic basal surface, it is very difficult to disperse natural talc in an aqueous medium, in ethanol (96%) and in dilute solutions of acids and alkali hydroxides. Although compounds such as nafion, carboxymethylcellulose (CMC) can increase the solubility of talc, allowing better adhesion of talc on a glassy carbon electrode for electroanalysis applications, our study aims at the possibility of using talc-like material without binder on a solid electrode. The nafion, a membrane and ion exchange resins, or CMC with numerous hydroxyl (-OH) functions and carboxyl groups of interest in electroanalysis [57,58,59,60,61,62,63], would automatically modify the electrochemical (ion exchange, charge transfer resistances) and electroanalytical properties (better accumulation of Pb^2+^ ions and consequently a better detection limit in aqueous solution) of talc, when the latter is used to prepare composites. Since these compounds do not solve the problem of hydrophobicity of talc and consequently difficulty to disperse it in water, while the synthesized amino-functionalized talc can be dispersed in aqueous solution and be stable on the glassy carbon electrode, we undertook to study its electrochemical and electroanalytical properties by comparing it to bare GCE. 

### 3.2. Electrochemical Characterization of Modified GCE by Cyclic Voltammetry

**Figure 6:**
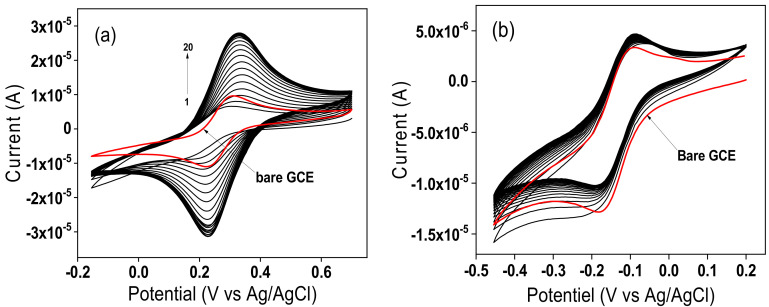
Multisweep cyclic voltammograms recorded in 0.1 M KCl containing (**a**) 1 mM [Fe(CN)_6_]^3−^ and (**b**) 1 mM [Ru(NH_3_)_6_]^3+^ on TalcNH_2_/GCE. The red curve in (**a**,**b**) corresponds to the probe signal recorded on the bare GCE, v = 50 mV/s.

The electrochemical characterization of the sensor consisting of the GCE modified with TalcNH_2_ clay (TalcNH_2_/GCE) was first performed in solution with neutral pH using cyclic voltammetry (CV). Negatively charged ([Fe(CN)_6_]^3−^) and positively charged ([Ru(NH_3_)_6_]^3+^) redox probes were probes were used to verify and confirm the ion exchange properties of TalcNH_2_. The analysis of [Fe(CN)_6_]^3−^ was carried out within a potential window ranging from −0.15 V to 0.7 V in 0.1 M KCl and the results are depicted on Figure 6a. As can be seen, the CV peak current of the first scan on TalcNH_2_/GCE (Ipa = 4.91 µA and Ipc = 5.15 µA) is lower compared to that observed on bare GCE (Ipa = 8.07 µA and Ipc = 8.93 µA). This is due to a gradual increase of ions on the electrode surface through the binding sites or pores of the electrode material. Not all [Fe(CN)_6_]^3−^ ion binding sites on the electrode surface have the same accessibility. An increase in CV peak currents is then observed up to the 20th scan (Ipa = 24.5 µA and Ipc = 23.5 µA). The high peak currents (anodic and cathodic) and a strong accumulation up to the 20th scan could be due to the electrostatic attraction between the negatively charged redox system [Fe(CN)_6_]^3−^ and the positively charged amino (-NH_3_^+^) groups present on the organosilane fragments used in the synthesis process. Several recently published works have highlighted the good affinity that protonated amine functions have in an acid medium to interact (through electrostatic interactions) with either negatively charged molecules or those having free pairs of electrons capable of reacting with other species in solution [64,65,66]. 

Similarly, the analysis of [Ru(NH_3_)_6_]^3+^ ions was carried out in the potential range from −0.6 V to 0.2 V in 0.1 M KCl, and the results are presented on Figure 6b. The comparison of the signals revealed that the oxidation and reduction peaks are more intense on the bare GCE (Ipa 5.490 µA, Ipc 6.187 µA) compared to TalcNH_2_/GCE (Ipa 2.110 µA, Ipc 2.164 µA). The lower peak current and non-accumulation obtained on TalcNH_2_/GCE could be explained by the fact that the synthesised and protonated TalcNH_2_ film in solution acted as an electrostatic barrier, preventing the absorption of the cationic species [Ru(NH_3_)_6_]^3+^. These electrochemical results support the results obtained during the physico-chemical characterisations which confirmed the effectiveness of the synthesis of a talc-like material and the incorporation of APTES into the structure of the synthesised product.

### 3.3. Determination of Electroactive Surface Area

**Figure 7:**
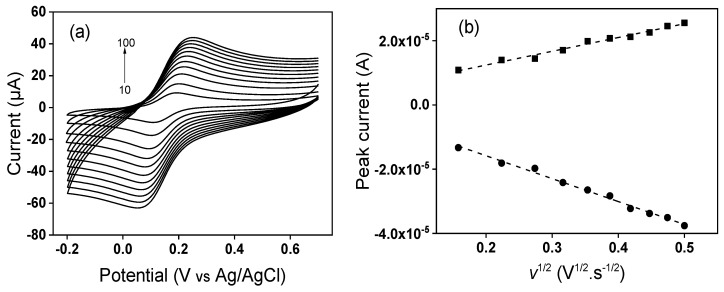
(**a**) Cyclic voltammograms at various scan rates (10 to 100 mV·s^−1^) recorded in 0.1 M KCl containing 1 mM [Fe(CN)_6_]^3−^ on bare GCE, and (**b**) linear plot of Ip (peak current) vs. *v*^1/2^.

**Figure 8:**
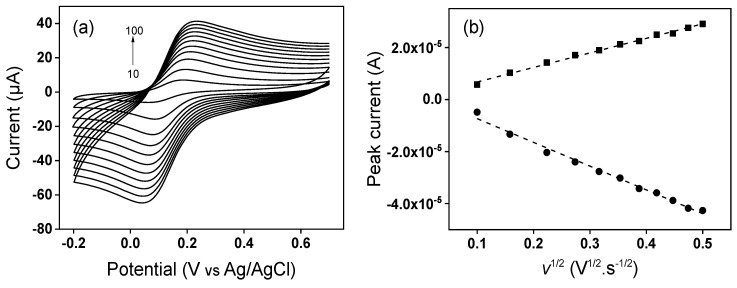
(**a**) Cyclic voltammograms at various scan rates (10 to 100 mV·s^−1^) recorded in 0.1 M KCl containing 1 mM [Fe(CN)_6_]^3−^ on TalcNH_2_/GCE, and (**b**) linear plot of Ip (peak current) vs. *v*^1/2^.

The electrochemically active surface areas of bare GCE and TalcNH_2_/GCE were estimated, using the [Fe(CN)_6_]^3−/4−^ redox system and applying the Randles–Sevcik for a reversible Equation (1):

(1) $$I_{p}\;=\;(2.69\;\times \;10^{5})An^{3/2}D^{1/2}CV^{1/2}$$

where *I*_p_ is the peak current, *A* the electrode electroactive area (cm^2^), *n* the number of electrons transferred, *D* the diffusion coefficient of [Fe(CN)_6_]^3−^ in a 0.1 M KCl solution, *C* the concentration of [Fe(CN)_6_]^3−^ (mol·cm^−3^) and *v* the potential scan rate (V·s^−1^) [67]. Cyclic voltammetry experiments at different scan rates, as shown on Figure 7 and Figure 8 were performed, and the obtained slopes of the Ip (peak current) vs. v^1/2^ plots for the [Fe(CN)_6_]^3−^ oxidation process were 4.29 × 10^−5^ A·v^−1/2^·s^1/2^ for the GCE and 5.59 × 10^−5^ A·v^−1/2^·s^1/2^ for the TalcNH_2_/GCE. From the *D* value for [Fe(CN)_6_]^3−^ equal to 7.6 × 10^−6^ cm^2^·s^−1^ [68], the corresponding electroactive areas were (0.058 ± 0.001) and (0.075 ± 0.002) cm^2^ for the GCE and TalcNH_2_/GCE, respectively. From these results, we can conclude that the electrochemical response of the [Fe(CN)_6_]^3−^ probe is affected by modification on the GCE, with the TalcNH_2_ material allowed an increase in the electroactive area by 1.3 fold compared to the GCE.

### 3.4. Impedance Characterization 

**Figure 9:**
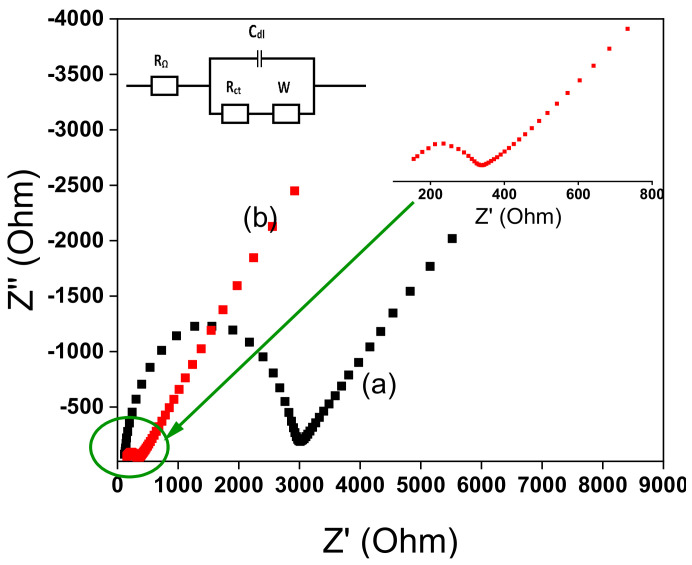
Electrochemical impedance spectroscopy (EIS) of bare GCE (**a**) and TalcNH_2_/GCE (**b**) in 0.1 M KCl containing 1 mM K_3_Fe(CN)_6_/K_4_Fe(CN)_6_. Frequency range: 0.01 Hz–10 kHz.

Using electrochemical impedance spectroscopy (EIS), it is possible to elucidate the heterogeneous electron transfer properties at the electrode-solution interface. The EIS curves display semi-circular and linear areas corresponding respectively to a process limited by electron transfer and diffusion. The diameter of semicircle is equal to the charge transfer resistance (*R*_ct_) [69]. Figure 9 shows the Nyquist plots recorded at bare GCE and TalcNH_2_/GCE in 0.1 M KCl containing 1 mM [Fe(CN)_6_]^3−/4−^. On the bare GCE, the value of Rct was 2725.7 Ω (Figure 9a). On TalcNH_2_/GCE, the value of Rct was decreased to 186.8 Ω (Figure 9b), which was much smaller than that of TalcNH_2_/GCE, indicating higher electron transfer for the TalcNH_2_-modified electrode. It was due to the presence of high conductive amino-synthetic material on GCE. As known, higher electron transfer and charge carrier density will increase the sensitivity of the target sensor. All these results indicated that Pb^2+^ ions can be successfully reduced on the surface of TalcNH_2_/GCE. The higher electrocatalytic behavior of the TalcNH_2_/GCE was confirmed by its lower charge transfer resistance. The EIS was also used to calculate the standard heterogeneous rate constant for the two electrodes in accordance with Equation (2) [50]:

(2) $$k^{o}\;=\;\;\frac{RT}{F^{2}R_{\mathrm{ct}}AC}$$

where *k*° is the standard heterogeneous electron transfer rate constant (cm·s^−1^), *R* the universal gas constant (8.314 J·K^−1^·mol^−1^), *T* the thermodynamic temperature (298.15 K), *F* the Faraday constant (96485 C·mol^−1^), *R*_ct_ the electron transfer resistance (Ω), *A* the electrode surface area (cm^2^) and *C* the concentration of the [Fe(CN)_6_]^3−/4−^ solution (10^−6^ mol·cm^−3^).

The *k*° values were obtained for the bare GCE (0.0017 ± 0.0003 cm·s^−1^) and TalcNH_2_/GCE (0.019 ± 0.001 cm·s^−1^). The *k*° values give information on the kinetic ease of a reaction process. A system with a low *k*° value will reach equilibrium faster than a system with a high *k*° value. The *k*° value is greater on TalcNH_2_/GCE than on GCE, indicating a faster electron transfer on this electrode, a property that is very beneficial in electrochemistry both in terms of energy savings and analysis time.

### 3.5. Electrochemical Behavior of Pb^2+^ Ions at TalcNH_2_/GCE

**Figure 10:**
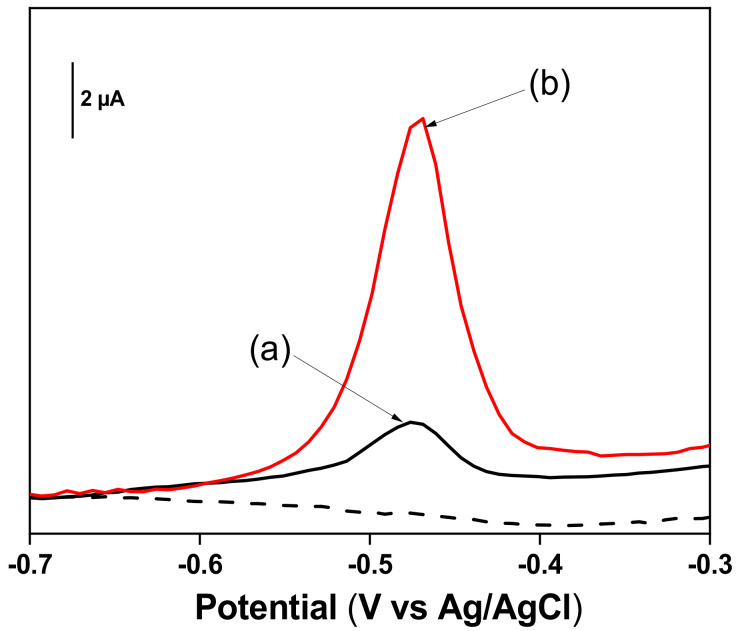
Anodic stripping differential pulse voltammetry (ASDPV) curves obtained on (**a**) GCE, and (**b**) TalcNH_2_/GCE of 5 µM Pb^2+^ in 0.1 M HCl after 30 s electrolysis at −0.8 V. Accumulation time: 1 min.

After the characterization of the TalcNH_2_ and the TalcNH_2_/GCE, the ability of the modified electrode to detect Pb^2+^ ions was investigated and preliminary experiments were carried out. The comparison between the anodic stripping differential pulse voltammetry responses on a bare GCE (curve a) and TalcNH_2_/GCE (curve b) is shown on Figure 10. It indicated that the sensitivity was higher on the TalcNH_2_/GCE. 

The results demonstrated that the peak height of Pb^2+^ ions on TalcNH_2_/GCE was about 3 times more important than that obtained on bare GCE. This shows the better affinity between Pb^2+^ ions and TalcNH_2_ synthesized clay which can be explained by the presence of amino functional groups -NH_2_ on its surface and in its structure that can easily chelate Pb^2+^ ions.

### 3.6. Optimization of the Experimental Conditions for the Detection of Lead Ions atTalcNH_2_/GCE

To achieve a best detection of Pb^2+^ ions with the TalcNH_2_ modified GCE, parameters such as pH of the accumulation medium, the concentration of the detection medium, the deposition potential and the deposition time were optimised.

**Figure 11:**
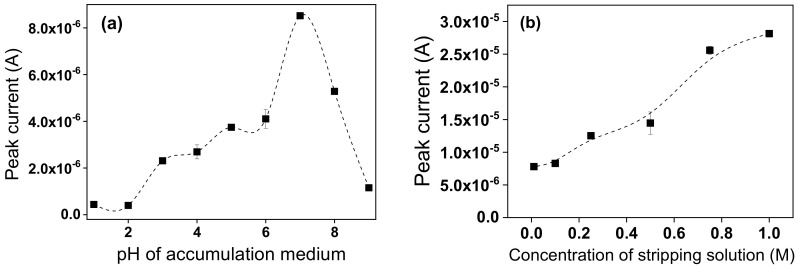

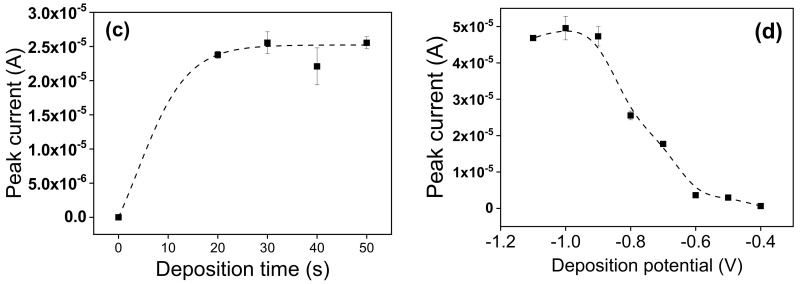
Influence of pH of the accumulation medium (**a**), the concentration of the stripping solution (**b**), the deposition time (**c**) and the deposition potential (**d**), on the stripping current response of TalcNH_2_/GCE (accumulation time of 1 min in 5 µM Pb^2+^). The experiments were conducted in triplicate.

The pH of the accumulation or detection medium can influence the electrochemical response of the modified electrodes with respect to the detection of heavy metal ions [70]. The ionization of functional groups on the TalcNH_2_ clay surface depends on the pH of the solution. At pH < pKa (the pKa of amines being around 9–10), most of these functional groups are mainly in ionized form (protonated amine) and can exchange H^+^ with metal ions in solution. The effect of the pH of the accumulation medium (from 1 to 9) on the peak current was studied in the range of 1.0 to 9.0, in order to find the right pH value to define the optimal values of detection of lead ions. The results were shown in Figure 11a.

The results (Figure 11a) showed the current peaks are low for pH values up 5.0 due to its mainly protonated state (protonated amine groups) of TalcNH_2_ material that can prevent the fixation of lead ions (predominant in accumulation medium) on its surface via electrostatic repulsions forces. The absorption of H^+^ (H^+^ coming from hydrochloric acid (HCl) used) hinders the adsorption of Pb^2+^, as it is a stronger acid than Pb^2+^. However, the peak currents increase when the pH increase from 1.0 to 7.0, and further decrease up to a pH value of 9.0. Yet, in the pH range 3.0–7.0, polymeric hydroxocomplexes of lead, mainly Pb(OH)^-^ and Pb(OH)_2_ predominate due to hydrolysis reactions [71], which inhibit the accumulation of lead ions [72]. The pH value 7 was chosen as optimal value for further studies. The pH range from 6.0 to 7.0 corresponds approximately to the beginning of the formation of the first lead monohydroxide complex Pb(OH)^+^ and to a low concentration of H^+^ in solution. Pb(OH)^+^ can therefore be easily absorbed by TalcNH_2_ when the concentration of H^+^ becomes much lower. 

The concentration of detection medium (H_3_O^+^, Cl^−^) was studied from 0.01 M to 1 M on TalcNH_2_/GCE and the results were presented in Figure 11b. As shown, the electrode response was less between 0.01 M and 0.1 M due to the smaller amount of H^+^ ions in the solution capable of weakening the complexes formed during the accumulation stage. After 0.1 M, the electrode response increased significantly and was more quantitative at 1 M. This may be due to the increase of H^+^ ions in solution. A concentration of 1 M of HCl was selected as the stripping medium for further studies. The deposition time was studied in the range of 0 to 45 s. The results in Figure 11c showed that the stripping current intensities of Pb^2+^ ions increased up to 30 s and almost reached the maximum at 30 s. Regarding sensitivity and measurement time in practice, 30 s was retained as the optimal deposition time for further investigations. To obtain the best sensitivity for TalcNH_2_/GCE, the deposition potential was studied from −0.4 to −1.1 V. The results presented in Figure 11d showed higher peak currents between −1.1 V and −0.9 V. The electrode response significantly decreased when the deposition potential shifted from −0.9 V to −0.4 V, due to lower amount of energy required to reduce Pb^2+^ ions. In the following study, −0.9 V was used as the optimal deposition potential. 

### 3.7. Detection Limit, Interference Study and Analytical Application of the Developed Sensor 

**Figure 12:**
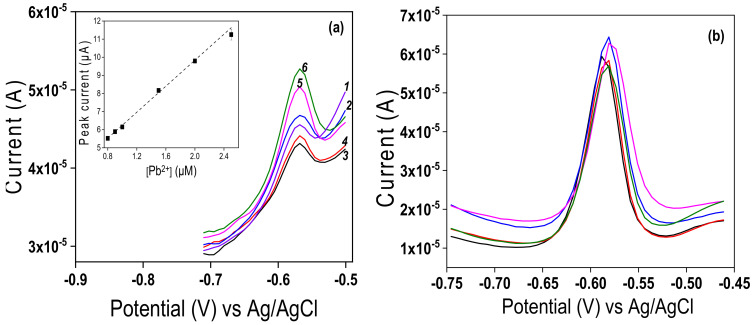
(**a**) Differential pulse anodic stripping voltammetry (DPASV) response of the TalcNH_2_/GCE at different concentrations of Pb^2+^ ions under optimal conditions (insert corresponding to calibration curve). The experiments were conducted in triplicate. (**b**) Series of 5 DPASV response of 5 µM Pb^2+^, recorded in 0.1 M HCl after 1 min accumulation in pH 6 aqueous solution.

Under the optimal conditions, Figure 12a showed that the anodic stripping peak currents of Pb^2+^ increased with concentration in the range of 0.8 µM to 2.5 µM (curve 1 to curve 6). The calibration curve (insert of Figure 12a) was linear over the studied concentration range, with the equation Ip(A) = 3.6[Pb^2+^](M) + (2.66 × 10^−6^) (R^2^ = 0.997). The detection limit (D_L_) and quantitation limit (Q_L_) were calculated from D_L_ = 3 S_b_/m [73,74], and Q_L_ = 10 S_b_/m, where S_b_ is the standard deviation of the blank and m is the slope of the calibration curve. D_L_ and Q_L_ were estimated to be 7.45 × 10^−8^ M and 24.84 × 10^−8^ M, respectively, and the sensitivity of the method of 3.6 µA.µM^−1^ was obtained. The performances of the sensor developed were compared with some other electrochemical sensors of Pb^2+^ ions (Table 2). Prior to the application of TalcNH_2_/GCE as a sensing device, the reproducibility of the electrode was checked by performing five successive measurements of 5 µM Pb^2+^ on different electrodes (GCE surface was renewed between successive runs). A relative standard deviation of 5.65% was obtained, showing the stability, repeatability and reproducibility of TalcNH_2_/GCE (Figure 12b).

**Figure 13:**
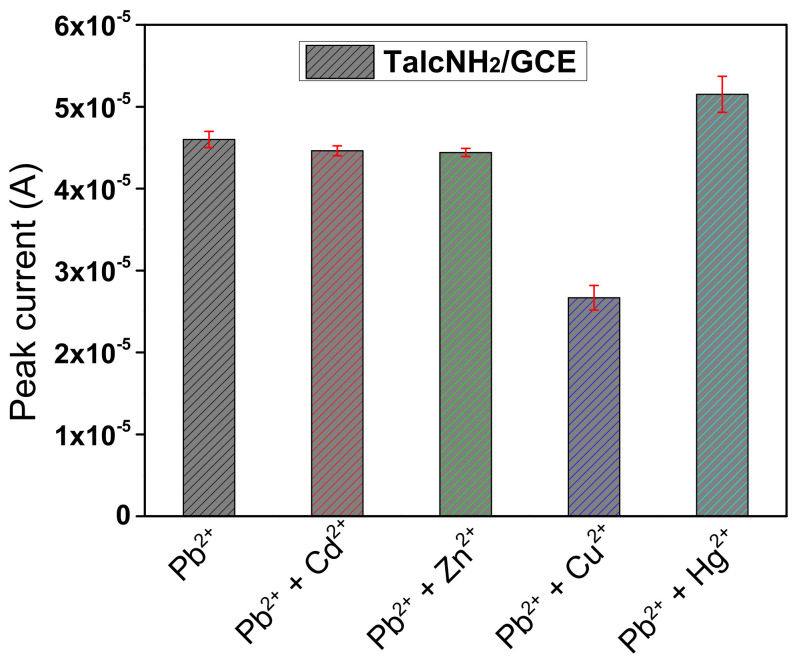
Interference studies of TalcNH_2_/GCE at 5 μM Pb^2+^ ions in the presence of 5 μM Cu^2+^, Cd^2+^, Zn^2+^ and Hg^2+^ ions under optimal experimental conditions. Experiments were performed in triplicate.

Figure 13 shows the DPASV responses toward 5 μM Pb^2+^ in the presence of 5 μM Cu^2+^, Cd^2+^, Zn^2+^ and Hg^2+^ under optimal experimental conditions. It was observed that the presence of Cd^2+^ and Zn^2+^ does not significantly influence the signal of Pb^2+^ ions. However, a decrease in the peak current of Pb^2+^ ions was observed upon addition of Cu^2+^, due to its high affinity for protonated amine groups on the synthesized TalcHN_2_. A significant increase in peak current was observed when Hg^2+^ were added, due to code position of Hg(0) during the detection step. It was found that some other ions, Na^+^, K^+^, Cl^−^, SO_4_^2−^ and NO_3_^−^, which were not listed in Figure 13, did not interfere with the signal of Pb^2+^.

The applicability of TalcNH_2_/GCE as a sensing device was evaluated for the detection of traces of lead in tap water sample. Tap water was used to prepare accumulation medium at a final Pb^2+^ concentration of 5 µM. After analysis under optimal conditions, a recovery amount of 95.65% of added Pb^2+^ ions was obtained. A high recovery obtained indicated that the TalcNH_2_/GCE can be successfully applied to analyse water polluted by lead ions.

## 4. Conclusions

Overall, this study has shown that it is possible to use a synthetic talc-type clay, easily functionalized with amine chelating groups as electrode material to improve the sensitivity and selectivity of the resulting modified electrodes. Indeed, a synthetic talc-like magnesium phyllosilicate amino-functionalized was obtained from the sol–gel process. Infrared spectroscopy, X-ray diffraction, CHN elemental analysis, SEM, BET method and UV–Vis diffuse reflectance spectroscopy study results confirmed the synthesis process. We have demonstrated that a TalcNH_2_/GCE can be used for the analysis of Pb^2+^ ions by stripping voltammetry. The ability of the TalcNH_2_ to complex Pb^2+^ ions onto the electrode was precise and accurate. After optimization of the factors affecting the pre-concentration and stripping steps, a detection limit of 7.45 × 10^−8^ M was obtained. The amino-functionalized talc/glassy carbon electrode prepared in this study was applied in the determination of Pb^2+^ ions in tap water with good results. This study demonstrates the potential of TalcNH_2_ synthetic material, a hydrophilic talc-like magnesium phyllosilicate amino-functionalized to serve as electrode material for the preparation of sensing devices dedicated to the detection of heavy metals.

## Figures and Tables

**Table 1:** Chemical composition of Nat-Talc and TalcNH_2_.

	Molar Ratios						
	Reaction Mixture		Product		Weight (%)		
	Si/Mg	C/N	Si/Mg	C/N	C	H	N
Nat-Talc	-	-	1.33	-	0.357	0.469	0
TalcNH_2_	1.33	3.00	0.50	3.33	14.90 (14.57 *)	4.83 (4.89 *)	4.42 (5.10 *)

* Theoretical values.

**Table 2:** Comparison of the performance of some Pb^2+^ ion sensors.

Electrode	D_L_ (µM)	Linear Range (µM)	Reference
Ca_10_(PO_4_)_6_(OH)_2_ modified CPE *	0.0768	0.002–0.24	[75]
Nanosized HA nafion modified GCE	0.001	0.005–0.8	[76]
GCE covered by engineered MWCNT **	0.0188	0.4–80	[77]
GCE covered by zinc oxide nanofibers functionalized by L-cysteine	0.0012	0.03–0.42	[78]
CPE modified with magnetic eggshell nanocomposite and MWCNT	0.452	1.5–600	[79]
TalcNH_2_/GCE	0.0745	0.8–2.5	This work

* CPE: Carbon paste electrode, ** MWCNT: Multi-walled carbon nanotubes.

## Data Availability

Not applicable.

## References

[1] Guggenheim S., Adams J.M., Bain D.C., Bergaya F., Brigatti M.F., Drits V.A., Formoso M.L.L., Galán E., Kogure T., Stanjek H. (2006). Summary of recommendations of nomenclature committees relevant to clay mineralogy: Report of the Association Internationale pour l’Etude des Argiles (AIPEA) Nomenclature Committee for 2006. Clays Clay Miner..

[2] Wang J., Somasundaran P. (2005). Adsorption and conformation of carboxymethyl cellulose at solid–liquid interfaces using spectroscopic, AFM and allied techniques. J. Colloid Interface Sci..

[3] Lugwisha E.H.J. (2009). Properties of fired bodies made from Tanzanian talc-clay mixes for ceramic applications. Tanzan. J. Sci..

[4] Ahmed M.M., Ibrahim G.A., Hassan M.M.A. (2007). Improvement of Egyptian talc quality for industrial uses by flotation process and leaching. Int. J. Miner. Process..

[5] Temuujin J., Okada K., Jadambaa T., Mackenzie K.J.D., Amarsanaa J. (2002). Effect of grinding on the preparation of porous material from talc by selective leaching. J. Mater. Sci. Lett..

[6] Sanchez-Soto P.J., Wiewióra A., Avilés M.A., Justo A., Pérez-Maqueda L.A., Pérez-Rodríguez J.L., Bylina P. (1997). Talc from Puebla de Lillo, Spain. II. Effect of dry grinding on particle size and shape. Appl. Clay Sci..

[7] Wallqvist V., Claesson P.M., Swerin A., Schoelkopf J., Gane P.A.C. (2009). Influence of Wetting and Dispersing Agents on the Interaction between Talc and Hydrophobic Particles. Langmuir.

[8] Da Fonseca M.G., Airoldi C. (2001). New amino-inorganic hybrids from talc silylation and copper adsorption properties. Mater. Res. Bull..

[9] Wesołowski M. (1984). Thermal decomposition of talc: A review. Thermochim. Acta.

[10] Feng B., Peng J., Guo W., Zhang W., Ai G., Wang H. (2018). The effect of changes in pH on the depression of talc by chitosan and the associated mechanisms. Powder Technol..

[11] Yi H., Zhao Y., Rao F., Song S. (2018). Hydrophobic agglomeration of talc fines in aqueous suspensions. Colloids Surf. A Physicochem. Eng. Asp..

[12] Boyd S.A., Lee J.-F., Mortland M.M. (1988). Attenuating organic contaminant mobility by soil modification. Nature.

[13] Da Fonseca M.G., Silva C.R., Airoldi C. (1999). Aminated Phyllosilicates Synthesized via a Sol–Gel Process. Langmuir.

[14] Fukushima Y., Tani M. (1995). An organic/inorganic hybrid layered polymer: Methacrylate–magnesium(nickel) phyllosilicate. J. Chem. Soc. Chem. Commun..

[15] Burkett S.L., Press A., Mann S. (1997). Synthesis, Characterization, and Reactivity of Layered Inorganic–Organic Nanocomposites Based on 2:1 Trioctahedral Phyllosilicates. Chem. Mater..

[16] Fonseca M.G., Airoldi C. (2000). Mercaptopropyl magnesium phyllosilicate—Thermodynamic data on the interaction with divalent cations in aqueous solution. Thermochim. Acta.

[17] Fukushima Y., Tani M. (1996). Synthesis of 2:1 Type 3-(Methacryloxy) propyl Magnesium (Nickel) Phyllosilicate. Bull. Chem. Soc. Jpn..

[18] Silva C.R., Fonseca M.G., Barone J.S., Airoldi C. (2002). Layered Inorganic–Organic Talc-like Nanocomposites. Chem. Mater..

[19] Da Fonseca M.G., Airoldi C. (2000). New layered inorganic–organic nanocomposites containing n-propylmercapto copper phyllosilicates. J. Mater. Chem..

[20] Da Fonseca M.G., da Silva Filho E.C., Machado Junior R.S.A., Arakaki L.N.H., Espinola J.G.P., Airoldi C. (2004). Zinc phyllosilicates containing amino pendant groups. J. Solid State Chem..

[21] Minet J., Abramson S., Bresson B., Sanchez C., Montouillout V., Lequeux N. (2004). New Layered Calcium Organosilicate Hybrids with Covalently Linked Organic Functionalities. Chem. Mater..

[22] Mizutani T., Fukushima Y., Okada A., Kamigaito O. (1990). Synthesis of nickel and magnesium phyllosilicates with 1:1 and 2:1 layer structures. Bull. Chem. Soc. Jpn..

[23] Sasai R., Itoh H., Shindachi I., Shichi T., Takagi K. (2001). Photochromism of Clay–Diarylethene Hybrid Materials in Optically Transparent Gelatin Films. Chem. Mater..

[24] Da Fonseca M.G., Silva C.R., Barone J.S., Airoldi C. (2000). Layered hybrid nickel phyllosilicates and reactivity of the gallery space. J. Mater. Chem..

[25] Toeri J., Osorio-Madrazo A., Laborie M.-P. (2017). Preparation and Chemical/Microstructural Characterization of Azacrown Ether-Crosslinked Chitosan Films. Materials.

[26] Järup L. (2003). Hazards of heavy metal contamination. Br. Med. Bull..

[27] Elliott P., Arnold R., Barltrop D., Thornton I., House I.M., Henry J.A. (1999). Clinical lead poisoning in England: An analysis of routine sources of data. Occup. Environ. Med..

[28] Anthemidis A.N., Ioannou K.-I.G. (2010). Development of a sequential injection dispersive liquid–liquid microextraction system for electrothermal atomic absorption spectrometry by using a hydrophobic sorbent material: Determination of lead and cadmium in natural waters. Anal. Chim. Acta.

[29] Wan Z., Xu Z., Wang J. (2006). Flow injection on-line solid phase extraction for ultra-trace lead screening with hydride generation atomic fluorescence spectrometry. Analyst.

[30] Hsieh H.-F., Chang W.-S., Hsieh Y.-K., Wang C.-F. (2009). Lead determination in whole blood by laser ablation coupled with inductively coupled plasma mass spectrometry. Talanta.

[31] Arnold M.A., Meyerhoff M.E. (1988). Recent Advances in the Development and Analytical Applications of Biosensing Probes. C R C Crit. Rev. Anal. Chem..

[32] Ngouoko J.J.K., Tajeu K.Y., Temgoua R.C.T., Doungmo G., Doench I., Tamo A.K., Kamgaing T., Osorio-Madrazo A., Tonle I.K. (2022). Hydroxyapatite/L-Lysine Composite Coating as Glassy Carbon Electrode Modifier for the Analysis and Detection of Nile Blue A. Materials.

[33] Sun D., Sun Z. (2008). Electrochemical determination of Pb2+ using a carbon nanotube/Nafion composite film-modified electrode. J. Appl. Electrochem..

[34] Yantasee W., Lin Y., Hongsirikarn K., Fryxell G.E., Addleman R., Timchalk C. (2007). Electrochemical Sensors for the Detection of Lead and Other Toxic Heavy Metals: The Next Generation of Personal Exposure Biomonitors. Environ. Health Perspect..

[35] Tonlé I.K., Letaief S., Ngameni E., Walcarius A., Detellier C. (2011). Square Wave Voltammetric Determination of Lead(II) Ions Using a Carbon Paste Electrode Modified by a Thiol-Functionalized Kaolinite. Electroanalysis.

[36] Ngassa G.B.P., Tonlé I.K., Walcarius A., Ngameni E. (2014). One-step co-intercalation of cetyltrimethylammonium and thiourea in smectite and application of the organoclay to the sensitive electrochemical detection of Pb(II). Appl. Clay Sci..

[37] Jiokeng S.L.Z., Dongmo L.M., Ymélé E., Ngameni E., Tonlé I.K. (2017). Sensitive stripping voltammetry detection of Pb(II) at a glassy carbon electrode modified with an amino-functionalized attapulgite. Sens. Actuators B Chem..

[38] Guenang L.S., Dongmo L.M., Jiokeng S.L.Z., Kamdem A.T., Doungmo G., Tonlé I.K., Bassetto V.C., Jović M., Lesch A., Girault H. (2020). Montmorillonite clay-modified disposable ink-jet-printed graphene electrode as a sensitive voltammetric sensor for the determination of cadmium(II) and lead(II). SN Appl. Sci..

[39] Sales J.A.A., Airoldi C. (2005). Calorimetric investigation of metal ion adsorption on 3-glycidoxypropyltrimethylsiloxane + propane-1,3-diamine immobilized on silica gel. Thermochim. Acta.

[40] Tanev P.T., Pinnavaia T.J. (1995). A Neutral Templating Route to Mesoporous Molecular Sieves. Science.

[41] Sales J.A.A., Petrucelli G.C., Oliveira F.J.V.E., Airoldi C. (2006). Some features associated with organosilane groups grafted by the sol–gel process onto synthetic talc-like phyllosilicate. J. Colloid Interface Sci..

[42] Ukrainczyk L., Bellman R.A., Anderson A.B. (1997). Template Synthesis and Characterization of Layered Al- and Mg-Silsesquioxanes. J. Phys. Chem. B.

[43] Whilton N.T., Burkett S.L., Mann S. (1998). Hybrid lamellar nanocomposites based on organically functionalized magnesium phyllosilicate clays with interlayer reactivity. J. Mater. Chem..

[44] Kamdem Tamo A., Doench I., Morales Helguera A., Hoenders D., Walther A., Madrazo A.O. (2020). Biodegradation of Crystalline Cellulose Nanofibers by Means of Enzyme Immobilized-Alginate Beads and Microparticles. Polymers.

[45] Marquez-Bravo S., Doench I., Molina P., Bentley F.E., Tamo A.K., Passieux R., Lossada F., David L., Osorio-Madrazo A. (2021). Functional Bionanocomposite Fibers of Chitosan Filled with Cellulose Nanofibers Obtained by Gel Spinning. Polymers.

[46] Lall A., Kamdem Tamo A., Doench I., David L., Nunes de Oliveira P., Gorzelanny C., Osorio-Madrazo A. (2020). Nanoparticles and Colloidal Hydrogels of Chitosan–Caseinate Polyelectrolyte Complexes for Drug-Controlled Release Applications. Int. J. Mol. Sci..

[47] Djouonkep L.D.W., Tamo A.K., Doench I., Selabi N.B.S., Ilunga E.M., Lenwoue A.R.K., Gauthier M., Cheng Z., Osorio-Madrazo A. (2022). Synthesis of High Performance Thiophene–Aromatic Polyesters from Bio-Sourced Organic Acids and Polysaccharide-Derived Diol: Characterization and Degradability Studies. Molecules.

[48] Deussi Ngaha M.C., Kougoum Tchieda V., Kamdem Tamo A., Doungmo G., Njanja E., Kenfack Tonle I. (2022). Aminoalcohol-functionalization of Alkali Palm Oil Fiber and Application as Electrochemical Sensor for 2-nitrophenol Determination. Electroanalysis.

[49] Osorio-Madrazo A., David L., Peniche-Covas C., Rochas C., Putaux J.-L., Trombotto S., Alcouffe P., Domard A. (2015). Fine microstructure of processed chitosan nanofibril networks preserving directional packing and high molecular weight. Carbohydr. Polym..

[50] Silverstein R.M., Webster F.X., Kiemle D.J. (2015). Spectrometric Identification of Organic Compounds.

[51] Pavia D.L., Lampman G.M., Kriz G.S. (1979). Introduction to Spectroscopy: A Guide for Students of Organic Chemistry.

[52] Nakamoto K. (2008). Infrared and Raman Spectra of Inorganic and Coordination Compounds.

[53] Wilson M.J. (1994). Clay Mineralogy: Spectroscopic and Chemical Determinative Methods.

[54] Fritsch E., Balan E., Petit S., Juillot F. (2021). Structural, textural, and chemical controls on the OH stretching vibrations in serpentine-group minerals. Eur. J. Miner..

[55] Dongmo L.M., Guenang L.S., Jiokeng S.L.Z., Kamdem A.T., Doungmo G., Victor B.C., Jović M., Lesch A., Tonlé I.K., Girault H. (2021). A new sensor based on an amino-montmorillonite-modified inkjet-printed graphene electrode for the voltammetric determination of gentisic acid. Mikrochim. Acta.

[56] Ferrage E., Martin F., Petit S., Pejo-soucaille S., Micoud P., Fourty G., Ferret J., Salvi S., de Parseval P., Fortune J.P. (2003). Evaluation of talc morphology using FTIR and H/D substitution. Clay Miner..

[57] Osorio-Madrazo A., Laborie M.-P., Dufresne A., Thomas S., Pothen L.A. (2013). Morphological and Thermal Investigations of Cellulosic Bionanocomposites. Biopolymer Nanocomposites.

[58] Von Palubitzki L., Wang Y., Hoffmann S., Vidal-Y-Sy S., Zobiak B., Failla A.V., Schmage P., John A., Osorio-Madrazo A., Bauer A.T. (2020). Differences of the tumour cell glycocalyx affect binding of capsaicin-loaded chitosan nanocapsules. Sci. Rep..

[59] Samyn P., Osorio-Madrazo A., Barhoum A., Bechelany M., Makhlouf A. (2018). Native Crystalline Polysaccharide Nanofibers: Processing and Properties. Handbook of Nanofibers.

[60] Lizundia E., Costa C.M., Alves R., Lanceros-Méndez S. (2020). Cellulose and its derivatives for lithium ion battery separators: A review on the processing methods and properties. Carbohydr. Polym. Technol. Appl..

[61] Abushammala H., Pontes J.F., Gomes G.H., Osorio-Madrazo A., Thiré R.M.S.M., Pereira F.V., Laborie M.-P.G. (2015). Swelling, viscoelastic, and anatomical studies on ionic liquid-swollen Norway spruce as a screening tool toward ionosolv pulping. Holzforschung.

[62] Wei J., Zhou Y., Lv Y., Wang J., Jia C., Liu J., Zhang X., Sun J., Shao Z. (2019). Carboxymethyl Cellulose Nanofibrils with a Treelike Matrix: Preparation and Behavior of Pickering Emulsions Stabilization. ACS Sustain. Chem. Eng..

[63] Bentley F.E., Passieux R., David L., Osorio-Madrazo A. (2022). Pure Chitosan Biomedical Textile Fibers from Mixtures of Low- and High-Molecular Weight Bidisperse Polymer Solutions: Processing and Understanding of Microstructure–Mechanical Properties’ Relationship. Int. J. Mol. Sci..

[64] Amine S., Montembault A., Fumagalli M., Osorio-Madrazo A., David L. (2021). Controlled Polyelectrolyte Association of Chitosan and Carboxylated Nano-Fibrillated Cellulose by Desalting. Polymers.

[65] Doench I., Torres-Ramos M.E.W., Montembault A., Nunes de Oliveira P., Halimi C., Viguier E., Heux L., Siadous R., Thiré R.M.S.M., Osorio-Madrazo A. (2018). Injectable and Gellable Chitosan Formulations Filled with Cellulose Nanofibers for Intervertebral Disc Tissue Engineering. Polymers.

[66] Kamdem Tamo A., Doench I., Walter L., Montembault A., Sudre G., David L., Morales-Helguera A., Selig M., Rolauffs B., Bernstein A. (2021). Development of Bioinspired Functional Chitosan/Cellulose Nanofiber 3D Hydrogel Constructs by 3D Printing for Application in the Engineering of Mechanically Demanding Tissues. Polymers.

[67] Opallo M., Lesniewski A. (2011). A review on electrodes modified with ionic liquids. J. Electroanal. Chem..

[68] Goyal R.N., Gupta V.K., Chatterjee S. (2010). Voltammetric biosensors for the determination of paracetamol at carbon nanotube modified pyrolytic graphite electrode. Sens. Actuators B Chem..

[69] Martin Santos A., Wong A., Araújo Almeida A., Fatibello-Filho O. (2017). Simultaneous determination of paracetamol and ciprofloxacin in biological fluid samples using a glassy carbon electrode modified with graphene oxide and nickel oxide nanoparticles. Talanta.

[70] Wang Y., Wang L., Huang W., Zhang T., Hu X., Perman J.A., Ma S. (2017). A metal–organic framework and conducting polymer based electrochemical sensor for high performance cadmium ion detection. J. Mater. Chem. A.

[71] Bouwe R.G.B., Tonle I.K., Letaief S., Ngameni E., Detellier C. (2011). Structural characterisation of 1,10-phenanthroline–montmorillonite intercalation compounds and their application as low-cost electrochemical sensors for Pb(II) detection at the sub-nanomolar level. Appl. Clay Sci..

[72] Wei Y., Gao C., Meng F.-L., Li H.-H., Wang L., Liu J.-H., Huang X.-J. (2012). SnO_2_/Reduced Graphene Oxide Nanocomposite for the Simultaneous Electrochemical Detection of Cadmium(II), Lead(II), Copper(II), and Mercury(II): An Interesting Favorable Mutual Interference. J. Phys. Chem. C.

[73] Ghoneim M.M., Hassanein A.M., Hammam E., Beltagi A.M. (2000). Simultaneous determination of Cd, Pb, Cu, Sb, Bi, Se, Zn, Mn, Ni, Co and Fe in water samples by differential pulse stripping voltammetry at a hanging mercury drop electrode. Anal. Bioanal. Chem..

[74] Ebunang D.V.T., Tajeu K.Y., Pecheu C.N., Jiokeng S.L.Z., Tamo A.K., Doench I., Osorio-Madrazo A., Tonle I.K., Ngameni E. (2022). Amino-Functionalized Laponite Clay Material as a Sensor Modifier for the Electrochemical Detection of Quercetin. Sensors.

[75] El Mhammedi M.A., Achak M., Chtaini A. (2009). Ca_10_(PO_4_)_6_(OH)_2_-modified carbon-paste electrode for the determination of trace lead(II) by square-wave voltammetry. J. Hazard. Mater..

[76] Pan D., Wang Y., Chen Z., Lou T., Qin W. (2009). Nanomaterial/Ionophore-Based Electrode for Anodic Stripping Voltammetric Determination of Lead: An Electrochemical Sensing Platform toward Heavy Metals. Anal. Chem..

[77] Li X., Zhou H., Fu C., Wang F., Ding Y., Kuang Y. (2016). A novel design of engineered multi-walled carbon nanotubes material and its improved performance in simultaneous detection of Cd(II) and Pb(II) by square wave anodic stripping voltammetry. Sens. Actuators B Chem..

[78] Oliveira V.H.B., Rechotnek F., da Silva E.P., Marques V.d.S., Rubira A.F., Silva R., Lourenço S.A., Muniz E.C. (2020). A sensitive electrochemical sensor for Pb^2+^ ions based on ZnO nanofibers functionalized by L-cysteine. J. Mol. Liq..

[79] Mohammadi S., Taher M.A., Beitollahi H. (2020). Synthesis and application of a natural-based nanocomposite with carbon nanotubes for sensitive voltammetric determination of lead (II) ions. Int. J. Environ. Anal. Chem..

